# The new wave of ocean industrialization and the challenges for biodiversity conservation in the Mediterranean Sea: the case of the Costa Brava

**DOI:** 10.1038/s41598-025-15279-z

**Published:** 2025-08-28

**Authors:** J. Lloret, P. Wawrzynkowski, R. Sardá, R. Rigall, A. Mujal-Colilles

**Affiliations:** 1 Institut de Ciències del Mar (ICM-CSIC), Pg. Marítim de la Barceneta 37-49, 08003 Barcelona, Spain; 2 Faculty of Earth Sciences, University of Barcelona, C/Martí i Franqués s/n, 08028 Barcelona, Spain; 3 Centre d’Estudis Avançats de Blanes (CEAB-CSIC), C/ d’accés a la Cala St. Francesc, 14, 17300 Blanes, Spain; 4 Faculty of Business and Economic Sciences, Universitat de Girona, C/ de la Universitat de Girona, 10, 17003 Girona, Spain; 5 Barcelona School of Nautical Studies, UPC-BarcelonaTECH, Pla de Palau, 18, 08003 Barcelona, Spain

**Keywords:** Ocean economy, Blue economy, Marine conservation, Maritime spatial planning, Ocean industrialization, Good environmental status, Marine biology, Environmental impact

## Abstract

To explore the new wave of ocean industrialization and the associated environmental challenges for biodiversity conservation in the Mediterranean Sea, we present here a case study of the Costa Brava region (northwestern Mediterranean), where conservation measures -particularly in areas of high ecological value-, are under increasing stress from new and emerging industrial activities. Using multiple data sources, and a spatiotemporal approach, this article considers the different economic activities in the study area and focuses on the various environmental impacts they may have. Fisheries and aquaculture landings, leisure boating infrastructure (berths), and cruise passenger activity exhibit particularly high levels both inside and near Marine Protected Areas (MPAs), as well as within or adjacent to other areas of conservation value. Notably, planned offshore wind farms and hydrogen pipelines are also located within or in close proximity to MPAs and other areas of conservation value. Our results indicate that the current Good Environmental Status (GES) of MPAs—a key concept describing ecosystems that are productive, resilient, and capable of sustaining both biodiversity and human activities—could be compromised by industrial activities, particularly those that have expanded in the region since the 2000s, including leisure boating, international cruising, and emerging industries such as offshore wind farms, hydrogen pipelines, and desalination plants.

## Introduction

The oceans are a developing and vitally important economic frontier, and the site of a new phase of industrialization. They are increasingly regarded as a resource that will satisfy present and future human needs for food, material, and space. As demand for marine resources and space continues to grow, expectations for the ocean as an engine of human development are increasing^1^. The ‘Blue acceleration’—a race among diverse and often competing interests for ocean food, material, and space—^1^ is gaining pace. In 2017, the global ocean economy generated estimated annual revenues of $5.2 trillion and provided employment for 168 million people, growing faster than the overall global economy^2^. However, while the economic benefits of the global ocean economy can be substantial, scientific evidence points to an alarming decline in ocean health^3^.

In the discourse on the global ocean economy, environmental concerns have historically been overlooked, with more emphasis being placed on the economic benefits of the industrial sectors involved^2^. However, as the focus shifted to meet this challenge, and to make the global economy more sustainable and restore ocean health, the concept of the Blue economy was introduced during the 2012 United Nations Rio + 20 Conference. The aim was to transform traditional marine activities and industries in order to promote equity and social well-being without compromising the ocean’s ecological integrity and its capacity to provide ecosystem goods and services^4^. The Blue economy concept gained traction in academic and policy circles, but today it has splintered and some have moved away from its central premise. In the context of this paper, we use the original concept of Blue economy, i.e. the one synonymous with a “Sustainable Ocean-Based Economy”^5^.

The global ocean economy encompasses both established sectors and emerging sectors^6,7^. Established sectors can involve living marine resources, non-living marine resources, conventional marine renewable energy, port activities, shipbuilding and repair, maritime transport and coastal and maritime tourism^6–9^. In the context of Europe, recent figures show these sectors directly employed close to 3.59 million people—with Spain being the largest employer—while generating around €623.6 billion in turnover, and €171.1 billion in gross value added^7^. On the other hand, the emerging and innovative sectors may involve more recent marine renewable energy technologies (e.g. development of floating wind and solar energy, tidal energy and offshore hydrogen generation), blue bio-economy and biotechnology (based on groups of marine organisms that are not traditionally exploited, and their biomass applications, for example, algae production), deep-water mining (exploitation of critical minerals), desalination, and recent efforts in restoration activities^7^.

In the context of an increasing demand for sea space by the industrial sectors and the resulting rise of anthropogenic pressures, Marine Protected Areas (MPAs) are becoming key elements of marine resource management and conservation (reviewed by^10,11^). Well-designed and well-managed MPAs can also provide a myriad of economic benefits through the services and goods they offer^10^. Although, in theory, MPAs can play a major role in reducing anthropogenic pressures, maintaining and improving biodiversity, building ecosystem resilience and providing socioeconomic benefits for coastal communities^12–14^, in practice, the many pressures from industrial sectors occurring inside or nearby protected areas can make it difficult to attain the desired goals^15–17^.

The marine ecoregions of the Mediterranean—despite numerous conservation initiatives—remain among the twenty most impacted ecoregions of the world^18^, with their marine biodiversity at major risk^19^. In this context, spatial prioritization for conservation areas is required to minimize negative pressures from areas of human activities and reduce conflicts between the two, with the goal of achieving effective conservation outcomes^19^. However, a lack of global spatio-temporal observational data limits our understanding of where and how the ocean economy is expanding^20^. Traditionally, interactions between ocean-based industrial sectors and conservation measures in the Mediterranean have been addressed in a sector-specific manner, without assessing potential cumulative spatial and temporal pressures. Moreover, these efforts have primarily focused on impacts within Natura 2000 sites, often overlooking other areas of conservation importance^55^. Hence, what is lacking is an integrated approach that evaluates the environmental effects of all industrial sectors and considers all types of areas of conservation value, whether they have regulatory power or not.

To explore the new wave of ocean industrialization in relation to biodiversity conservation in the Mediterranean Sea, we introduce here, using multiple data sources and Geographical Information Systems (GIS) complemented with temporal analysis of data and a literature review, a case study of the Costa Brava region (Catalonia, Spain, North Western Mediterranean). To the best of our knowledge, this study represents the first attempt in the Mediterranean Sea to identify the potential pressures that all industrial oceanic sectors may have on all areas critical for marine biodiversity conservation, regardless of their legal status, using a spatio-temporal approach (i.e. mapping the interactions between economic sectors and areas and analyzing the trends in activity of these sectors). The aim of this interdisciplinary study is to assess how large-scale industrialization of the sea, if not correctly planned and managed, can alter ocean health and biodiversity conservation objectives, as well as the expectations of stakeholders and society regarding the territory, and its associated visions. This paper intends to support decision-making by providing greater clarity on the spatial aspects of this new wave of industrialization in the Mediterranean, triggered by the expanding industrial sectors, in order to ensure sustainable outcomes and to foster an evidence-based assessment that balances the economic benefits of these activities with the environmental costs they incur. To do so, we first assessed the spatial overlap of traditional and emerging industrial activities with MPAs—specifically, Natura 2000 sites, which are backed by regulation, and other areas of marine conservation value that are not legally designated to protect marine biodiversity and not officially recognized as MPAs (Supplementary Table S1). The possible effects of the different industrial sectors on these marine areas important for biodiversity conservation are dealt with by examining the magnitude of each activity inside, or bordering, the areas. Second, we conducted a temporal analysis of each sector to determine changes in activities over time (2000–2023). Third, we evaluated the pressures of these sectors on different components of the marine environment and their corresponding changes in state in terms of Good Environmental Status (GES), a key concept used mainly in marine environmental policy, especially under the European Union’s Marine Strategy Framework Directive (MSFD). It defines a state in which marine ecosystems are productive, resilient, and capable of sustaining biodiversity and human activities. Finally, based on the outcomes, we discuss the environmental and economic trade-offs between the industrial activities and the conservation measures. This paper aims to help decision making by providing greater clarity on the spatial aspects of this new wave of industrialization in the Mediterranean Sea.

## Materials and methods

### Study area

**Fig. 1:**
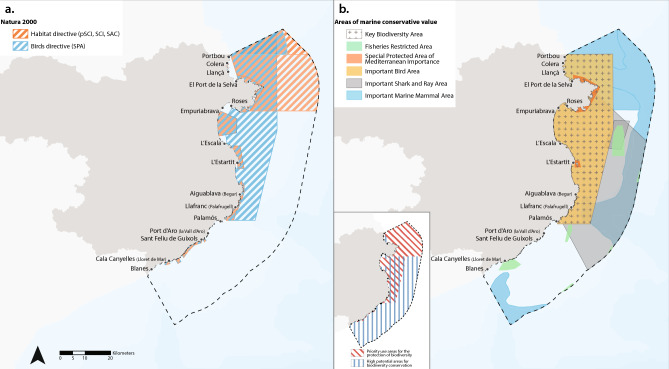
Maps of MPAs and other areas of marine conservation value in the Costa Brava region. (**a**) Natura 2000 protected sites: Sites of Community Importance (SCI), Special Areas of Conservation (SAC), Special Protection Areas (SPA), and proposed sites of community importance (pSCI); (**b**) Other areas of conservation value: Key Biodiversity Areas (KBA), Fisheries Restricted Areas (FRA), Specially Protected Areas of Mediterranean Importance (SPAMI), Important Bird Areas (IBA), Important Shark and Ray Areas (ISRA), Important Mammal Areas (IMMA), and Priority Areas for the Conservation of Biodiversity and Areas of High Potential for the Conservation of Biodiversity set up in the Maritime Spatial Planning (MSP) plans developed by the Spanish government^34^.

The Costa Brava stretches from the French/Spanish Border to the city of Blanes in the province of Girona (Catalonia, Spain), in the northwestern Mediterranean, consisting of approximately 220 km of coastline (Fig. 1). It comprises three different counties (Alt Empordà, Baix Empordà and La Selva). It is an important tourist and fishing region, in which the ocean economy generates a high economic impact, with about €560 million in revenues (data for the year 2021) involving around 600 companies, directly or indirectly creating thousands of jobs in different sectors^21^. The Costa Brava region shares its northern border with the Gulf of Lion, one of the 18 Mediterranean areas identified as priority areas for immediate conservation action^19^. The area has a rich marine biodiversity, with productive and diverse ecosystems, such as seagrass meadows, coralligenous assemblages, and submarine canyons^22–27^. The natural value of this wide diversity of habitats has prompted the declaration of a good number of MPAs^28^. The area considered in this study lies within Spanish territorial waters (from the shoreline to 12 nautical miles offshore). The socioeconomic and environmental characteristics of the Costa Brava allow us to classify this region into two of the four homogeneous environmental management units (HEMUs) obtained in a previous work related to planning of marine and coastal activities in Catalonia^29^. More specifically, the marine region of the Alt Empordà county is classified as a ‘highly natural’ area, whereas the marine regions of the two other counties of the Costa Brava (Baix Empordà and La Selva) are classified as ‘seminatural’ areas^29^.

### Mapping and assessing the trends in the ocean economy sectors

We used GIS to map the ocean economy sectors. All maps were created using the ArcGIS Pro software^30^. We considered the established and emerging sectors in the study area, all of which meet two criteria: (a) they are marine-based sectors as defined in the latest EU Blue Economy Report^7^ and (b) they have certain industrial features, in line with Smith^31^, in that they require high levels of investment and technology, and are associated with large-scale infrastructure. Following these criteria, the five sectors we analyzed were: (i) fisheries and aquaculture, (ii) marine energy, including associated infrastructure such as electric substations, submarine cables and pipelines, (iii) maritime transport of goods, (iv) marine and coastal tourism (leisure boating and cruises), and (v) desalination. In our analyses, we have not included marine-related activities that use and/or produce goods and services for ocean and marine-based activities (for example, seafood processing, shipbuilding and repair, port activities, maritime telecommunication, or maritime insurance), nor have we considered other activities driven mainly by the public sector (e.g. maritime defense and surveillance, marine conservation, education and research, and coastal engineering, such as dredging for beach nourishment and flood defense work). In most sectors, data disaggregated by categories are largely unavailable. For example, total landings are reported in official statistics without distinguishing between different fishing fleet types, such as small-scale fisheries and industrial trawlers. Where disaggregated data were available—such as for cruise tourism—we used them to analyze data separately for small (local) and large (international) cruises. In the case of leisure boating, although more than 80% of the fleet consists of vessels under 10 m in length, we classified it as an industrial activity due to its large-scale footprint in the area and the substantial infrastructure it requires (e.g., marinas and ports).

Data were compiled from various sources on seafood tonnage—originating from both fisheries and aquaculture—landed at fishing ports within the study area, along with the corresponding revenues generated. Additional information collected includes the number of fishing vessels, details of aquaculture facilities, the volume of goods transported in and out of Costa Brava ports, passenger traffic (distinguishing between local and international cruises), and recreational marine activities. Additionally, data used to generate the map of total port revenues derived from these sectors were also gathered. Detailed information on data sources can be found in Supplementary Table S2. We developed a percentage-based distribution of human activities across areas to explore the potential relationship between activity concentration and associated environmental and economic impacts, assuming that higher activity levels indicate greater potential impacts.

For mapping energy proposals, the Offshore Wind Development Area (OWDA)—where multiple offshore wind farms and one pilot project have been proposed by energy developers and public authorities—as well as the planned cable route transmitting electricity from the OWDA to the mainland, and the map of the proposed submarine hydrogen, were obtained from different sources detailed in Supplementary Table S2.

For mapping maritime traffic, we used publicly available Automatic Identification System (AIS) data, which records vessel coordinates to monitor movement and enhance maritime safety (see Supplementary Table S2 for details). However, due to the fact that small recreational vessels are not required to carry AIS devices, it was not possible to generate a route density map for this category. To evaluate the route densities of different vessel types (fishing vessels, cargo vessels, tankers, and cruise ships) inside and outside MPAs)—distinguishing between Natura 2000 sites and MPAs without regulatory enforcement—data were tested for normality using the Shapiro–Wilk test. Fishing activity in the analysis included only vessels over 24 m in length and those employing active gear, as these are required to carry AIS devices. Small-scale fishing vessels were excluded due to the lack of AIS data or any other tracking device. All datasets failed the normality test (p < 0.01), indicating non-normal distribution. Consequently, the non-parametric Mann–Whitney U test (W) was applied to compare route densities between protected and non-protected areas, as well as between areas inside and outside other zones of conservation importance. We assumed that higher vessel density is associated with greater environmental impact, although this relationship has not been consistently demonstrated across all sectors (see ‘Limitations of the Study’ in the Discussion section for further details). To analyze trends from 2000 to 2023 across various sectors and variables—namely fisheries and aquaculture, total revenues, maritime transport of goods, and tourism—regression analyses were performed using the Ordinary Least Squares (OLS) model with the *statsmodels* Python package. Figures displaying the 95% confidence intervals were generated using the *seaborn* data visualization library.

### Mapping marine protected areas (MPAs)

We identified and mapped (Fig. 1a) the legally designated MPAs in the study area, specifically the Natura 2000 sites, using the European Environmental Agency Natura 2000 database (https://www.eea.europa.eu/). Natura 2000 is the name of the ecological network of protected sites for selected habitats and species within the EU, which includes Sites of Community Importance (SCI), proposed sites of community importance (pSCI), and Special Areas of Conservation (SAC) that meet the Habitats Directive (Habitats 92/43/EEC Directive); and Special Protection Areas (SPA) that meet the Birds Directive (Birds 2009/147/EC Directive). The Natura 2000 sites analyzed here are those encompassing marine areas. All maps were created using the ArcGIS Pro software^30^. Overall, a large portion of the study area is protected: Natura 2000 protected sites encompass 44% of the study area’s surface.

### Mapping other areas of marine conservation value

Areas of conservation value that are not legally designated to protect marine biodiversity and not officially recognized as MPAs were identified and mapped (Fig. 1b). These included (i) areas designated under the Barcelona Convention for the Protection of the Mediterranean Sea against Pollution (the so-called Specially Protected Areas of Mediterranean Importance, SPAMI), (ii) areas recommended by different institutions, such as Important Bird Areas (IBA), Important Marine Mammal Areas (IMMA), Key Biodiversity Areas (KBA), and Important Shark and Ray Areas (ISRA), and (iii) Fisheries Restricted Areas (FRA), which, although backed by Spanish legislation, have fisheries sustainability as their primary goal rather than biodiversity conservation. Some of these areas overlap with Natura 2000 sites. All areas were mapped using shape files found in public repositories or provided by the organizations responsible for the designation of these areas, as was the case with the IMMAs and KBAs^32,33^. Finally, we mapped the Priority Areas for the Conservation of Biodiversity and the Areas of High Potential for the Conservation of Biodiversity set up in the Maritime Spatial Planning (MSP) plans developed by the Spanish government^34^ (Fig. 1b). Although all these areas remain non-protected, they possess outstanding biodiversity values, making them strong candidates for future designation as legally protected MPAs. Overall, these areas occupy 77% of the study region, some of which overlap the Natura 2000 sites. There are no “Other Effective Area-based Conservation Measures” (OECMs) defined within the study area.

### Pressures of industrial activities on Good Environmental Status (GES) of MPAs

A review of the peer-reviewed and grey literature was undertaken to qualitatively assess the pressures of the industrial sectors on different components of the marine environment of Mediterranean MPAs, and their corresponding impacts in terms of Good Environmental Status (GES). We used the Web of Science platform as the primary database to search and source publications, with the main search terms being various combinations of the different sectors and GES descriptors (Supplementary Table S3): Biodiversity, Non-indigenous species, Commercial fish and shellfish, Food webs, Eutrophication, Sea-floor integrity, Hydrographical conditions, Contaminants in the marine environment, Contaminants in seafood, Marine litter, and Energy, including Underwater Noise. We did not follow a formal systematic review process, as our aim was to identify key studies illustrating the overall impacts of sectors on GES. The only papers chosen, listed in Supplementary Table S3, were those considered to be highly relevant with specific examples of the issues identified. Accordingly, we employed a narrative review approach for this component of the study, enabling a broad synthesis of key literature relevant to the topic. We could not carry out this review analysis with specific reference to the different area categories analyzed (MPAs and other areas of marine conservation value) because the information on such effects is not available for each category separately.

## Results

### Interaction of industrial sectors with marine areas important for biodiversity conservation

**Fig. 2:**
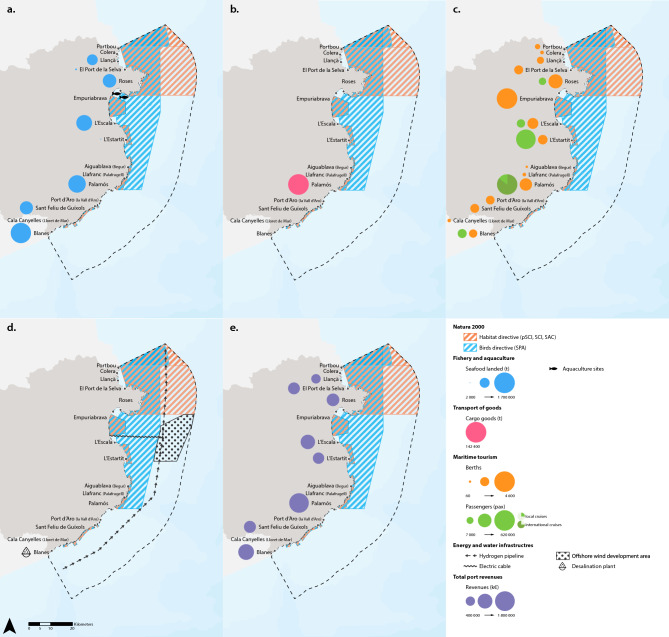
Maps of interaction of different ocean economy sectors with Natura 2000 protected sites: Sites of Community Importance (SCI), Special Areas of Conservation (SAC), Special Protection Areas (SPA), and proposed sites of community importance (pSCI). (**a**) Fisheries and aquaculture: Seafood landed in fishing ports and aquaculture sites; (**b**) Transport of goods: Goods embarked and disembarked from cargo vessel; (**c**) Maritime tourism: Number of passengers embarked or disembarked on cruises and number of berths for recreational vessels; (**d**) Energy and water infrastructure: the hydrogen pipeline, the offshore wind development area (with the electric cable), and the desalination plant; (**e**) Total revenues by port. The main ports where the activities take place are shown. The colored circles represent the magnitude of each variable shown on the maps. Data for the year 2023.

**Fig. 3:**
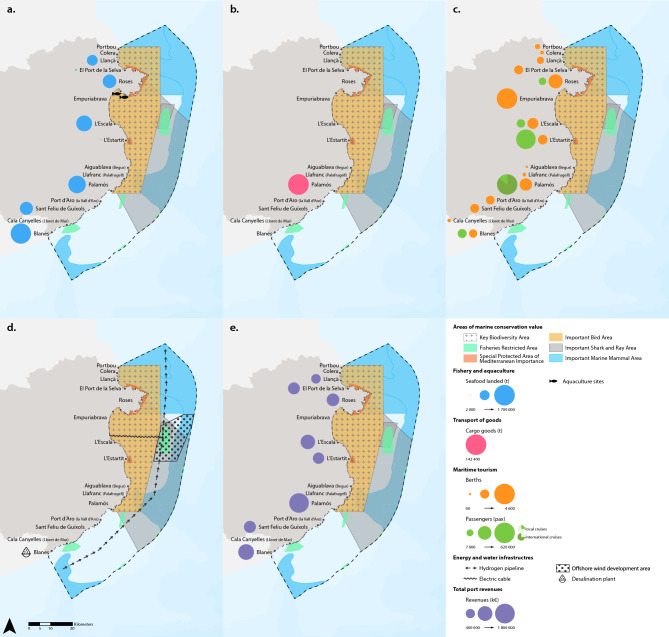
Maps of interaction of different ocean economy sectors with other areas of marine conservation value: Key Biodiversity Areas (KBA), Fisheries Restricted Areas (FRA), Specially Protected Areas of Mediterranean Importance (SPAMI), Important Bird Areas (IBA), Important Shark and Ray Areas (ISRA) and Important Mammal Areas (IMMA). (**a**) Fisheries and aquaculture: Seafood landed in fishing ports and aquaculture sites; (**b**) Transport of goods: Goods embarked and disembarked from cargo vessels; (**c**) Maritime tourism: Number of passengers embarked or disembarked on cruises and number of berths for recreational vessels; (**d**) Energy and water infrastructure: the hydrogen pipeline, the offshore wind development area (with the electric cable), and the desalination plant; (**e**) Total revenues by port. The main ports where the activities take place are shown. The colored circles represent the magnitude of each variable shown on the maps. Data for the year 2023.

Figures 2 and 3 show the 2023 maps of interactions between various industrial sectors (fisheries and aquaculture, maritime tourism, transport of goods, offshore wind energy, hydrogen pipelines, and desalination plants) and Natura 2000 protected sites—areas endowed with regulatory authority—as well as other marine conservation areas that are not legally designated to protect marine biodiversity.

The spatial interconnection of the different industrial sectors with these MPAs and other areas of conservation value (which is dealt with in detail in the following sections) is strong. Furthermore, the entire Costa Brava region is covered by a Priority Area for the Conservation of Biodiversity or an Area of High Potential for the Conservation of Biodiversity following the Spanish MSP plans^34^, after Directive 2014/89/EU, establishing a framework for MSP (Fig. 1b), and therefore, all of the industrial sectors considered here interact with one or both of these important conservation areas. The relevance of these conservation areas for the economy of the region is highlighted by the fact that, in 2023, 58% of the total port revenues occurred in ports situated in the vicinity (2 km buffer) of Natura 2000 protected sites, while 74% of the revenues occurred in ports situated inside or in the vicinity (2 km buffer) of other areas of marine conservation value (Table 1).

**Table 1:**
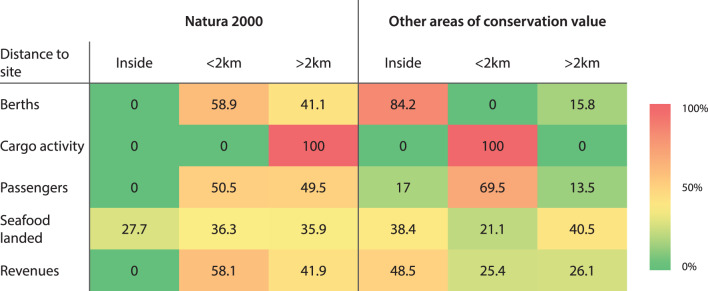
Percentage-based distribution of human activities across spatial areas. Seafood landed (tonnes), cargo activity (tonnes), passenger numbers (all cruises), number of leisure boat berths, and total port revenues (thousand euros) in Costa Brava ports categorized by distance to Natura 2000 protected sites and other marine conservation areas: inside, outside within 2 km, and outside beyond 2 km.

#### Fisheries and aquaculture

28% of seafood landings occur in fishing ports located inside Natura 2000 sites, 36% in a < 2 km buffer of a Natura 2000 site, and 36% of landings occur in fishing ports located at > 2 km from a Natura 2000 site (Table 1). On the other hand, 38% of seafood landings occur in ports that are inside other areas of conservation value, 21% occur in ports that are located < 2 km from these areas, with the remaining 40% occurring in ports that are located > 2 km from these other areas (Table 1). There are two aquaculture facilities: one located within a Natura 2000 protected site and the other outside it (Fig. 2a). Both facilities are situated within other areas of marine conservation value (Fig. 3a).

**Fig. 4:**
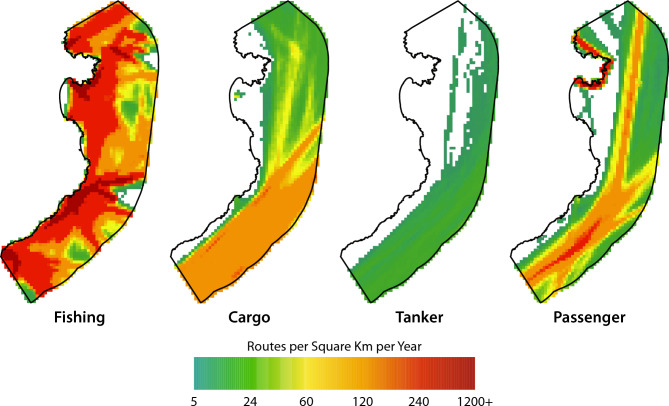
Maps of route densities of different vessel types (fishing vessels, cargo vessels, tankers, and cruises) in the Costa Brava region (all areas combined: Natura 2000 sites and other areas of conservation value). Densities are expressed as average presence of vessels in hours in a square kilometer per month (2023 averages).

Regarding fishing route densities (2023 averages), shown in Fig. 4, significant differences exist between densities inside Natura 2000 MPAs and non-protected areas (W = 1 113 777, p < 0.01), with higher values found inside Natura 2000 protected sites (mean = 284 h of fishing vessel in a square kilometer per month, sd = 318) than in non-protected areas (mean = 244 h of fishing vessel in a square kilometer per month, sd = 326). The difference in fishing route densities between areas of marine conservation value and surrounding waters was also statistically significant (W = 520,131, p < 0.01). In this case, lower densities were observed inside these areas (mean = 251 vessel hours/km^2^/month, sd = 314) compared to outside them (mean = 311 vessel hours/km^2^/month, sd = 351). The magnitude of the differences in both cases is small, as indicated by the low r^2^ values (r^2^ = 0.139 and r^2^ = 0.106 respectively).

#### Transport of goods

Cargo vessels load and unload their cargoes at only one port, Palamós, which is located > 2 km away from a Natura 2000 site and < 2 km from other areas of conservation value (Table 1). Tanker vessels do not disembark their liquids or gases in any port of the Costa Brava region.

Regarding cargo route densities (2023 averages), as shown in Fig. 4, significant differences are observed between routes inside and outside Natura 2000 MPAs (W = 267,512, p < 0.01) with lower values found inside them (mean = 27.5 h of cargo vessels in a square kilometer per month, sd = 26.9) than in non-protected areas (mean = 90.7 h of cargo vessels in a square kilometer per month, sd = 44.1). The magnitude of the difference is large (r^2^ = 0.62). Differences between other areas of marine conservation importance and surrounding waters were statistically significant (W = 237,062, p < 0.01), with lower cargo route densities inside these areas (mean = 53.41 vessel hours/km^2^/month, sd = 45.1) compared to outside (mean = 94.0 vessel hours/km^2^/month, sd = 48.70). However, the effect size was modest (r^2^ = 0.28), indicating a relatively small magnitude of difference.

Regarding tanker route densities (2023 averages) shown in Fig. 4, significant differences exist between route densities inside Natura 2000 and non-protected areas (W = 237 062, p < 0.01) with lower values found inside them (mean = 2.75 h of tanker vessel in a square kilometer per month, sd = 3.06) than in non-protected areas (mean = 14.90, sd = 9.31). The magnitude of the difference is large (r^2^ = 0.652). Similarly, significant differences were found between areas of marine conservation value and surrounding waters (W = 360,299, p < 0.01), with lower tanker route densities inside these areas (mean = 7.74 vessel hours/km^2^/month, sd = 8.62) compared to outside (mean = 15.10 vessel hours/km^2^/month, sd = 9.74). However, the magnitude of the difference is not very large (r^2^ = 0.28).

#### Maritime tourism

Table 1 shows that, on average in 2023, 59% of leisure boating berths were located in ports and marinas situated < 2 km from Natura 2000 protected sites, with the remaining 41% at > 2 km from them. On the other hand, 84% of berths were located in ports and marinas situated inside the other areas of marine conservation value, with the remaining 16% at > 2 km from them (Table 1). In addition, 50.5% of passengers were embarked or disembarked in ports that are located < 2 km from Natura 2000 sites, with the remaining 49.5% at > 2 km from these protected sites (Table 1). While for the other areas of conservation importance, 17% of passengers were embarked or disembarked in ports situated inside them, 69% of passengers at a distance < 2 km, and 13% in ports located > 2 km from them.

Regarding cruise route densities (2023 averages) (Fig. 4), the W tests indicated that significant differences exist between densities inside Natura 2000 protected areas and those inside non-protected areas (W = 509 770, p < 0.01) with lower values found inside Natura 2000 areas (mean = 47.41, sd = 97.10) than inside non-protected areas (mean = 68.22 h of cruises in a square kilometer per month, sd = 62.01). The magnitude of the difference is large (r^2^ = 0.40). A similar pattern was observed for cruise route densities inside and outside other areas of marine conservation value: differences between these areas and surrounding waters were statistically significant (W = 430,902, p < 0.01), with lower densities inside (mean = 51.91 vessel hours/km^2^/month, sd = 85.12) compared to outside (mean = 75.35 vessel hours/km^2^/month, sd = 59.90). However, the effect size was relatively small (r^2^ = 0.21), indicating a modest magnitude of difference.

#### Energy and water infrastructure

All the planned energy infrastructures, whether offshore wind farms or hydrogen pipelines, are located inside or within < 2 km of Natura 2000 sites and other areas of marine conservation value—in some cases, coinciding with their physical borders, as is the case with the offshore wind farms (Figs. 2d and 3d). The desalination plant (located in Blanes), with a current capacity of desalination of 20 Hm^3^, is located > 2 km away from Natura 2000 sites and other areas of conservation value (Figs. 2d and 3d). This plant, constructed in 2002 to alleviate the water supply deficit in this region, takes seawater via 10 beach wells located a few meters inland on the shoreline at the Tordera River Delta.

### Trends in industrial sectors

**Fig. 5:**
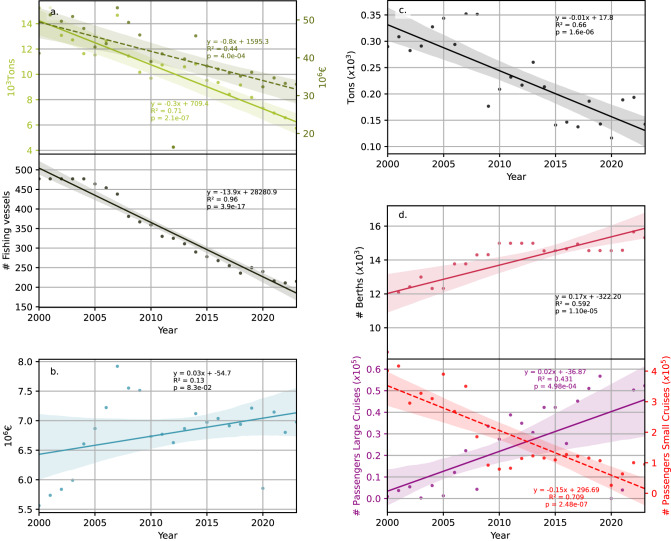
Trends in various ocean economic sectors in the Costa Brava ports between 2000 and 2023. (**a**) Fisheries and aquaculture: Annual landings (in tonnes and thousand euros, inflation adjusted) of fisheries and aquaculture products, number of fishing vessels; (**b**) Total revenues (in thousands of euros, inflation adjusted); (**c**) Maritime transport of goods: goods loaded and unloaded by cargo ships (in tonnes); (**d**) Tourism: number of berths for recreational vessels and number of passengers embarked and disembarked on small (local) and large (international) cruises The 95% confidence intervals are shown.

There is a downward trend for the period 2000–2023 in seafood landings in both tonnage and value in euros (inflation adjusted) (Fig. 5a), as well as in the number of fishing vessels (Fig. 5a), cargo activity (Fig. 5c), and the number of passengers embarked or disembarked on small (local) cruises (Fig. 5d). In contrast, in the same period, there was a rise in the number of berths for leisure boats (Fig. 5d), the number of passengers embarked or disembarked on large (international) cruises (Fig. 5d), and the total revenues in ports (Fig. 5b). Another upward trend concerns the capacity of desalination plants (Hm^3^ of water desalinated), which increased from zero Hm^3^ in 2000 to 20 Hm^3^ in 2023. Finally, there is a proposal for the construction of a 450 km hydrogen pipeline and up to 1205 MW of offshore wind power—the largest wind farm submitted for approval within the offshore wind development area—in the Costa Brava region.

### Potential impacts of industrial sectors on the Good Environmental Status (GES) of MPAs and other areas of conservation value

Supplementary Table S3 summarizes the potential environmental impacts of the industrial sectors considered in this study on Natura 2000 MPAs and the various areas of conservation importance, in relation to the 11 GES descriptors of the European Marine Strategy Framework Directive. All industrial sectors can potentially affect the GES of these valuable areas, with evidence of negative effects on biodiversity, seafloor integrity, non-indigenous species and contaminants in the environment being the most established. As shown in this table, the reviewed literature indicates that, overall, the industrial sectors analyzed can exert wide-ranging ecological impacts. These include habitat degradation, species overexploitation, biodiversity loss, alterations in food webs, eutrophication, and the introduction of non-indigenous species. These impacts are mediated through various stressors such as underwater noise and vibrations, collisions with fauna, water pollution, ballast water discharge, anchoring, and the introduction of artificial substrates, among others (Supplementary Table S3). Several of these stressors are cumulative—for example, noise and pollution from shipping, recreational boating, commercial fishing, cruising, and offshore wind energy. In contrast, the ecological effects of hydrogen pipelines and desalination plants remain largely unknown (Supplementary Table S3).

## Discussion

This study highlights strong spatial overlaps between key industrial sectors and MPAs and other areas of marine conservation value in the northwestern Mediterranean. Economic activity is closely linked to these areas, with over half of the total port revenues in the region generated within or near (within 2 km) MPAs. Fisheries and aquaculture landings, leisure boating infrastructure (berths), and cruise passenger activity exhibit particularly high levels both inside and near MPAs, as well as within or adjacent to other areas of conservation value. Notably, planned energy developments, including offshore wind farms and hydrogen pipelines, are also located within or in close proximity to MPAs. By contrast, cargo activity, along with desalination infrastructure, are predominantly located outside MPAs. Regarding vessel traffic, only large fishing vessels using active gears exhibited significantly higher densities within MPAs compared to areas outside them.

Trends from 2000 to 2023 show declines in fishing activity (reflected by decreasing seafood landings, revenues, and the number of fishing vessels), cargo transport, and local (small) cruise passengers, alongside increases in recreational boating, international cruise traffic, total port revenues, and desalination capacity. Additionally, the analyzed data indicate the potential future development of energy projects such as hydrogen pipelines and offshore wind farms. The findings also point to widespread impacts from these industrial activities across multiple GES descriptors. This suggests that the integrity of MPAs and other conservation zones—cornerstones for achieving GES, defined as a state in which marine ecosystems are healthy, productive, and resilient enough to support both biodiversity and human use—could be compromised by industrial activities. These impacts are particularly associated with sectors that have expanded in the region since the 2000s, such as leisure boating and international cruising, as well as emerging energy and water industries, including offshore wind farms, hydrogen pipelines, and desalination plants.

### The relevance of Mediterranean MPAs in the context of expanding sea industrialization

The industrialization of the seas began in the sixteenth and seventeenth centuries with the development of fisheries, commercial and defense shipping, and the conversion of coastal sites for shipbuilding and processing. This process has continued over the centuries, expanding to include coastal and marine tourism, mineral and energy extraction, and, more recently, renewable energy development and marine biotechnology^31^. Although the industrialization of the seas was once primarily limited to coastal areas, technological advances over the past decades have made even the most remote parts of the ocean accessible^1,35^.

As ocean industrialization continues to expand—driven by growth in sectors such as those analyzed in this study—MPAs have become increasingly important tools for ensuring that economic development does not come at the expense of marine ecosystem health^36,37^ Marine conservation through MPAs should be a top priority in light of the biodiversity crisis that is severely affecting marine ecosystems^36^. This is particularly important for Spain which, at 23%, is among the 20 countries in the world with the highest share of fragile ecosystems in the Biodiversity and Ecosystem services index^38^. It is equally important for the entire Mediterranean Sea, a hot spot for marine biodiversity^39^ and one which is threatened by human activities and global change^40^. All around the world, maritime infrastructure and traffic (shipping, cruising, and recreational boating) are growing and expanding rapidly, whereas professional fishing is in decline^20,37,41–44^. These trends are found in our study region: professional fishing and local cruise activity have declined since the early 2000s, whereas leisure boating, international cruise activity, water infrastructure (desalination plants) and energy infrastructure (offshore wind farms and hydrogen pipelines) have increased or are expected to increase dramatically in the future, driving the next phase of industrialization in the north-western Mediterranean Sea and raising new red flags for MPAs. A recent review of interactions between 14 ocean sectors revealed that 13 sectors (all of which have been included in our study) had interactions that produced unidirectional, negative ecosystem impacts^45^, the one exception being the telecommunications sector. Another study described the pressures imposed by 17 industrial sectors on GES areas, which represent the ultimate objective of the Marine Strategy Framework Directive (2008/56/EC)^2^. In this context, it is of paramount importance to protect the existing MPAs from further industrialization.

The three counties of the Costa Brava region, which comprise around 38% of the Catalan coastline, are responsible for about 35% of the non-market ecosystem service value of the Catalan coast^29,46^, highlighting the considerable economic value delivered to local citizens by adjacent protected ecosystems. In Europe as a whole, ecosystem services from Natura 2000 protected sites (terrestrial and aquatic combined) are estimated to be worth between 223 and 314 billion euros^47,48^. Although the initial economic costs of MPAs may outweigh any immediate economic benefits (because they often impose new management on marine activity by restricting or altering their existing spatial distribution), over the long-term, MPAs can make an important contribution to these maritime activities (reviewed by^10^). In this context, there is a need for further analyses of environmental and socioeconomic trade-offs among multiple and equally important environmental, economic, and social objectives, such as biodiversity conservation, marine renewables expansion, the protection of small-scale fishery livelihoods, sustainable aquaculture production, and food and nutritional security—not all of which may be fully reconciled^49–51^.

MPAs play a major role in shaping and defining seascapes by conserving ecological integrity and guiding sustainable human activities^36,37^. Pörtner et al.^52^ proposed a mosaic of interconnected protected and shared spaces, including intensively used spaces, to strengthen self-sustaining biodiversity, the capacity of people and nature to adapt to and mitigate climate change, and nature’s contributions to people. Maritime planning should embrace, maintain and respond to this logic. Mediterranean areas such as the Costa Brava, which were classified as a combination of highly natural and seminatural areas by Brenner et al.^29^, could meet the criteria of two of the various distinct ‘scapes’ defined by Pörtner et al.^52^ for Earth’s major biomes, namely “Large intact natural areas” (strictly protected areas and large, intact wilderness spaces assimilable to protected areas) and “Varied mosaic of nature and people” (spaces shared by people and biodiversity). However, the new wave of industrialization in the Mediterranean Sea, beginning in the early 2000s, but part of an ongoing industrialization of the world’s oceans during the second half of the 21st Century^31^, may transform these ‘scapes’ into “Heavily modified anthromes” (areas with cities, intensive fisheries, modified coast and energy infrastructure)^52^. If this happens, the United Nations’ goal to protect 30% of the world’s oceans by 2030, agreed upon by 196 countries and bodies such as the European Commission, will be very difficult to achieve.

### Upscaling of the results

The outputs of our study can be scaled-up. They can contribute to the process of weighing up the economic benefits of ocean activities against the environmental costs associated with them, as well helping decision-makers by providing greater clarity on spatial aspects of ocean economy sectors and marine conservation, and ensuring sustainable outcomes and equitable sharing of benefits with coastal communities. Our study has been carried out on the local scale and therefore reflects local realities and challenges more specifically than larger (national or sea basin) analyses, and this can be particularly useful for planning ocean economic activities^53^. MSP is usually conducted on a broad scale^54,55^, and often fails to avoid or minimize the conflicts that arise between local communities and industrial activities operating, or projected to operate, inside or nearby Mediterranean MPAs (see e.g.,^16,55,56^). In this context, the outputs of our interdisciplinary study favor a more tailored and localized approach to the planning of industrial activities, and one that must consider not only marine protected areas (Natura 2000 sites) but also other areas of conservation importance and a temporal approach (not only spatial) of human activity at sea.

### Beyond the planning of maritime activities

Beyond managing industrial activities to avoid damage to MPAs, an equitable downscaling of production and consumption that increases human well-being and enhances ecological conditions has also been proposed as a new vision for the seas to ensure not only environmental, but also social sustainability^57,58^. Market-based values are the core drivers of today’s global biodiversity crisis, and their primacy in many decisions needs to be reduced and balanced with the non-market instrumental, relational, and intrinsic values that are integral to why nature matters to people^59^. In order to incorporate nature’s diverse values into decision-making and to leverage transformative changes toward more just and sustainable futures, combinations of value-centered approaches are needed^59^. Industrialization can affect territorial visions and policies, which fall into three categories: anthropocentric (prioritizing human interests), biocentric-ecocentric (emphasizing living beings or nature’s processes as a whole) and pluricentric (encompassing all such views with no single ‘centre’)^59^. Depending on the number and type of companies and industries that begin to operate in a particular site, and the degree to which they attract other activities associated with them, the mix of these industrial sectors in a particular regional environment can alter a region’s vision for the future and the policies being pursued. This trend, widely studied on land, is now also accelerating in the marine environment and careful planning is needed to deal with it. In addition, safeguarding ocean sustainability requires transdisciplinary efforts to manage the activities of the many stakeholders, including governments, corporations, and civil society, and guide them toward responsible ocean stewardship^1^.

### Policy implications

As the expansion of human activity into the ocean accelerates, the marine natural systems, and the goods and services that they provide, deteriorate^2,3^. In past decades, the economic development of countries was mostly measured by industrialization targets and environmental consequences were ignored^60^. Such polices now require urgent re-examination to avoid further environmental deterioration, and to stay in line with international recommendations such as the Kunming-Montreal Global Biodiversity Framework^61^ and the 14th Sustainable Development Goal (SDG): “Conserve and sustainably use the oceans, seas and marine resources for sustainable development”^62^. This is especially important when new industrial activities are contemplated in areas that have hitherto been dedicated mostly to biodiversity conservation, or others that remain in relatively in good health. The Costa Brava region, 44% of whose marine territorial waters (Spanish) are protected, is a good example of this. Furthermore, other areas of conservation value—such as IMMA, ISRA, KBA, and IBA (Supplementary Table S1)—which together cover 77% of the study area, are currently unprotected but possess outstanding biodiversity value. These areas are strong candidates for future MPAs and should therefore be carefully considered in marine spatial planning—not just the Natura 2000 sites. MPAs will play an important role in the EU’s Biodiversity Strategy for 2030, which aims to effectively protect 30% of European seas by 2030, with one-third (10%) under strict protection^63^. However, in the Mediterranean Sea, just 9.7% of EU waters are currently covered by Natura 2000 sites, compared to 27.6% in the Greater North Sea and 15.5% in the Baltic Sea^64^. On the other hand, the EU biodiversity strategy for 2030 also recognizes that besides biodiversity conservation objectives, there is a need to bring substantial health, social and economic benefits to coastal communities, and the EU as a whole. In this sense, many studies have shown that MPAs can benefit coastal communities by helping professional fishing and local tourism business in a number of different ways (reviewed by^10,12,13,65^). Indeed, our study showed that around 60% of the total port revenues in the study area (for the year 2023) occurred in ports located inside or in the vicinity (2 km buffer) of Natura 2000 sites, and approximately 75% of total revenues occurred in ports located inside or in the vicinity of the other MPA categories, demonstrating that substantial economic value was delivered to local citizens by the MPAs surrounding them. Our results suggest that industrialization must be avoided, or limited, in the case of coastal regions that currently have the highest environmental values, and large areas of protected waters or areas of high conservation value. In particular, emerging industrial activities such as offshore wind energy should be prohibited within or adjacent (a buffer zone of at least 10 km should be maintained, Brill et al.^66^) to MPAs, with this restriction extended to all zones designated for marine biodiversity conservation^67^.

The management and conservation of the world’s oceans require a synthesis of spatial data on the distribution and intensity of human activities, and the overlap of their impacts on marine ecosystems^18^. One of the fundamental tools for managing spatial data and achieving a balance between human activities and biodiversity conservation in the EU and its Member States is MSP^68,69^. However, in many Mediterranean countries, MSP is still in its infancy^55^ and where MSP exists, it presents several challenges^55^. For example, the recently approved Spanish Maritime Spatial Plans^34^ revealed an extensive overlap between industrial sectors and important marine conservation areas, illustrating the challenges marine biodiversity conservation in Spain faces due to the expanding industrial sectors of the ocean economy. Early mapping of current and projected interactions between industrial activities and all types of areas of conservation value—regardless of their regulatory status—is essential for understanding and managing human activities that may impact ecologically important areas.

Contrary to the Precautionary Principle, industrial activities are, at times, expanding ahead of exploration, with licenses granted before a consensus is reached on how to mitigate the environmental impacts of the activity^1^. This also goes against the idea of using an Ecosystem-Based Approach (EBA), an important underlying principle within MSP, to achieve a sustainable use of the marine environment, and ultimately contribute to healthy seas across Europe^53^. This occurs because key principles that typically define the EBA—such as the application of the Precautionary Principle, holistic consideration of all ecosystem components, and the involvement of relevant stakeholders—are often not upheld when there is urgency to develop certain sectors, such as offshore wind energy, where the need to address climate change and energy security tends to accelerate development processes^28,70^. Furthermore, given the poor environmental status prevailing in parts of the European seas—such as sectors of the Baltic and North Sea Exclusive Economic Zones—implementing an EBA that supports the achievement and maintenance of GES will require a substantial reduction in the adverse impacts on marine ecosystems resulting from both existing and planned human uses of maritime space^71^. Apart from the ecological impacts described, industrial activities have major socioeconomic consequences, often at the expense of coastal communities^1^. Many coastal communities are excluded from the high-level decision-making processes that define some industrial sectors, such as renewable and non-renewable energy projects, cruising and shipping^31,72,73^. These large-scale businesses are associated with the exclusion of coastal communities that rely on small-scale, local businesses such as fishing and coastal tourism^20,31,73^. Benefits disproportionately flow to economically powerful states and corporations, whereas the harms are largely affecting developing nations and local communities^1,74^, which can have severe consequences for the health and well-being of coastal communities that depend on healthy seas^75,76^. These failures can be avoided or better addressed when policies align with a more comprehensive suite of biodiversity goals and local citizen’s values. Ignoring or marginalizing locally-held values in planning activities can leave a legacy of mistrust, and create conflicts with local (coastal) communities, jeopardizing program outcomes over time^15,77^.

Finally, policymakers and spatial planners should consider the impacts that certain industrial activities may have on the seascape and landscape of Mediterranean MPAs and other marine conservation areas, such as those in the Costa Brava. These areas provide significant scenic, amenity, and socioeconomic value to local and tourist communities who enjoy their views^78^, and their appreciation—along with other leisure and sport activities within marine parks—is often enhanced by the surrounding natural scenery^79^. For instance, the offshore wind industry, as a large-scale industrial enterprise, has the potential to significantly transform the seascape^80,81^. Consequently, the economic and social losses related to the degradation of seascapes in Mediterranean MPAs—key components of marine goods and services—caused by offshore wind farms could be substantial^28^.

### Gaps, limitations and future research

Despite the insights provided by this study, several gaps and limitations remain. Our study reveals substantial gaps in understanding the environmental pressures exerted by maritime infrastructures and activities on MPAs and other marine areas in the Mediterranean that serve conservation functions but lack legal designation. In particular, the impacts of hydrogen pipelines and water desalination plants remain poorly quantified, with offshore wind farms also requiring further study. Our study demonstrates that, as ocean industrialization accelerates through the expansion of these sectors, there is an urgent need to assess their environmental impacts on MPAs and other areas of high conservation value. Understanding these pressures reveals another important gap: the lack of natural capital metrics to evaluate GES in MPAs and other conservation areas. GES is a normative conceptual framework currently applied at broad geographic scales defined by individual countries; in our case study, this corresponds to the Levantino-Balear region as defined in the Spanish MSP plans^34^. Currently, GES indicators are only applicable at broad geographic scales, which complicates their application to specific conservation areas such as MPAs. Effective planning and management of human activities in these areas would be significantly improved if GES could be downscaled to the level of individual MPAs, supported by clearly defined indicators and metrics.

Our study has certain limitations, particularly the inability to analyze trends across different size categories and types of activity within each economic sector, due to data constraints—specifically, the lack of detailed information on the intensity of use within each sector. For instance, within the fishing sector, the ecological footprint of small-scale professional and recreational fishing fleets is generally considered to be lower than that of industrial trawling^82^. Similarly, the ecological impact of non-motorized craft (e.g., sailing boats and kayaks) is typically less significant than that of motorized vessels (e.g., yachts and jet skis)^41^. These distinctions are important and should be addressed in future research as more detailed and disaggregated data become available.

Another limitation of this study is that, for all sectors considered, a direct link between vessel density and environmental impact has not always been clearly established. For example, while there is a well-documented body of literature demonstrating a direct relationship between fishing effort density and habitat impact (e.g.,^83^), as well as between shipping route density and the risk of wildlife collisions^84^, the evidence remains more limited for other sectors such as cargo and passenger transport. In these cases, studies linking vessel density to specific ecological impacts remain relatively scarce or indirect, highlighting the need for further research.

## Conclusions

The growing influx of new industrial sectors such as offshore marine energy, hydrogen pipelines, and desalination plants, combined with the ongoing growth of the tourism sector (international cruises and leisure boating) is driving a new wave of industrialization in the Mediterranean This paper documents extensive impacts from industrial sectors across multiple Good Environmental Status (GES) indicators -a key concept representing a condition in which marine ecosystems are productive, resilient, and capable of sustaining both biodiversity and human activities -of Marine Protected Areas (MPAs) and other areas of marine conservation value-, including biodiversity loss, seafloor habitat degradation, the spread of invasive species, and increased pollution levels. In this context, our results indicate that industrialization should be avoided in Mediterranean coastal regions with high environmental value—such as the Costa Brava—which encompass extensive protected waters and other areas of significant conservation value. Additionally, the findings of our study underscore the need for a more tailored and localized approach to the planning of industrial activities—one that considers not only areas of conservation value beyond Natura 2000 sites, but also the temporal dimensions of human activity at sea.

## Supplementary Information

Supplementary Table S1.

Supplementary Table S2.

Supplementary Table S3.

## Data Availability

The datasets used and/or analysed during the current study available from the corresponding author on reasonable request.

## References

[1] Jouffray, J.-B., Blasiak, R., Norström, A. V., Österblom, H. & Nyström, M. The blue acceleration: the trajectory of human expansion into the ocean. *One Earth.* **2**, 43–54. 10.1016/j.oneear.2019.12.016 (2020).

[2] Sardá, R. et al. Business for ocean sustainability: Early responses of ocean governance in the private sector. *Ambio*10.1007/s13280-022-01784-2 (2023). 36260251 10.1007/s13280-022-01784-2 PMC9755432

[3] Duarte, C. M. et al. Rebuilding marine life. *Nature* **580**, 39–51. 10.1038/s41586-020-2146-7 (2020). 32238939 10.1038/s41586-020-2146-7

[4] United Nations. United Nations Conference Sustainable Development (UNCSD). Blue Economy Concept Paper. Johannesburg (Rio+20), (2012).

[5] Sardá, R., Pogutz, S., Theodorou, N. A. & Ramakrishna, K. The blue economy: What it is, what is not, and how to get there. In *Handbook of Sustainable Blue Economy* (eds Leal Filho, W. et al.) 10.1007/978-3-031-32671-4_48-1 (Springer, 2025).

[6] EU 2020. The EU Blue Economy report. 10.2771/363293.

[7] EU 2024. The EU Blue Economy report 10.2771/186064.

[8] OOF. *Business for Ocean Sustainability: A Global Perspective* (One Ocean Foundation, 2020).

[9] The Economist. The blue economy Growth, opportunity, and a sustainable ocean economy. Economist Intelligence Unit briefing paper for the World Ocean Summit 2015. (2015).

[10] ICF-IEEP-PML. Study on the Economic Benefits of MPAs Final Report. https://op.europa.eu/en/publication-detail/-/publication/dbe3d250-b0b5-11e8-99ee-01aa75ed71a1 (2018).

[11] SIMWESTMED. Supporting Implementation of Maritime Spatial Planning in the Western Mediterranean region. European Commission, Directorate-General for Maritime Affairs and Fisheries, Grant Agreement: EASME/EMFF/2015/1.2.1.3/02/SI2.742101 (2018).

[12] Costello, M. Evidence of economic benefits from marine protected areas. *Sci. Mar.* **88**(1), e080 (2024).

[13] Grorud-Colvert, K. et al. The MPA guide: A framework to achieve global goals for the ocean. *Science* **373**, eabf0861 (2021). 34516798 10.1126/science.abf0861

[14] Oldekop, J. A., Holmes, G., Harris, W. E. & Evans, K. L. A global assessment of the social and conservation outcomes of protected areas: social and conservation impacts of protected areas. *Conserv. Biol.* **30**, 133–141 (2016). 26096222 10.1111/cobi.12568

[15] Cánovas-Molina, A. & García-Frapolli, E. Untangling worldwide conflicts in marine protected areas: five lessons from the five continents. *Mar. Policy* **121**, 104185. 10.1016/j.marpol.2020.104185 (2020).

[16] Gómez, S., Carreño, A. & Lloret, J. Cultural heritage and environmental ethical values in governance models: Conflicts between recreational fisheries and other maritime activities in Mediterranean marine protected areas. *Mar. Policy*10.1016/j.marpol.2021.104529 (2021).

[17] WWF. Nature protection and offshore renewable energy in the Eurpean Union. Position paper. WWF European Policy Office. May 2021 (2021).

[18] Halpern, B. S. et al. A global map of human impact on marine ecosystems. *Science* **319**, 948–952 (2008). 18276889 10.1126/science.1149345

[19] Micheli, F. et al. Setting priorities for regional conservation planning in the Mediterranean Sea. *PLoS ONE* **8**(4), e59038. 10.1371/journal.pone.0059038 (2013). 23577060 10.1371/journal.pone.0059038 PMC3618442

[20] Paolo, F. S. et al. Satellite mapping reveals extensive industrial activity at sea. *Nature* **625**, 85–91. 10.1038/s41586-023-06825-8 (2024). 38172362 10.1038/s41586-023-06825-8 PMC10764273

[21] Cambres de Comerç de Girona, Palamós i Sant Feliu de Guíxols. Impacte socioeconòmic de l’Economia Blava de la Costa Brava. Report (in Catalan) (2022).

[22] Ballesteros, E., Mariani, S., Cefalí, M. E., Terradas, M. & Chappuis, E. Manual dels hàbitats litorals de Catalunya. Edited by Generalitat de Catalunya. (2014).

[23] Demestre, M. et al. Desvelando los paisajes submarinos. Editorial CSIC. ISBN 978-84-1352-844-1 (2023) (in Spanish).

[24] Dominguez-Carrió, C. et al. Diversity, structure and spatial distribution of megabenthic communities in Cap de Creus continental shelf and submarine canyon (NW Mediterranean). *Prog. Oceanogr.*10.1016/j.pocean.2022.102877 (2022).

[25] Gili, J. M. et al. Caracterización física y ecológica del área marina del Cap de Creus. Informe final área LIFE+ INDEMARES (LIFE07/NAT/E/000732). Instituto de Ciencias del Mar/CSIC (Barcelona) (in Spanish) (Coordinación: Fundación Biodiversidad, 2011).

[26] Lo Iacono, C., Orejas, C., Gori, A., Gili, J. M., Requena, S., Puig, P., & Ribó, M. Habitats of the Cap de Creus continental shelf and Cap de Creus Canyon, Northwestern Mediterranean. In *Seafloor Geomorphology as Benthic Habitat: GeoHab Atlas of Seafloor Geomorphic Features and Benthic Habitats* (eds Harris, P. & Baker, E.) 457–469. 10.1016/B978-0-12-385140-6.00032-3 (Elsevier, 2012).

[27] Sardá, R., Rossi, S., Martí, X. & Gili, J. M. Marine benthic cartography of the Cap de Creus (NE Catalan Coast, Mediterranean Sea). *Sci. Mar.* **76**(1), 159–171. 10.3989/SCIMAR.03101.18D (2012).

[28] Lloret, J. et al. Unravelling the ecological impacts of large-scale offshore wind farms in the Mediterranean Sea. *Sci. Total Environ.* **10**(24), 153803. 10.1016/j.scitotenv.2022.153803 (2022). 10.1016/j.scitotenv.2022.153803 35150689

[29] Brenner, J., Jiménez, J. A. & Sardá, R. Definition of homogeneous environmental management units for the Catalan coastal zone. *Environ. Manag.* **38**, 993–1005. 10.1007/s00267-005-0210-6 (2006). 10.1007/s00267-005-0210-6 16990984

[30] Esri. ArcGIS Pro (Version 3.1.0) [Software]. (Environmental Systems Research Institute, Inc., 2024).

[31] Smith, H. D. The industrialization of the world ocean. *Ocean Coast. Manag.* **43**, millenium essay, 11–28 (2000).

[32] BirdLife International. World database of key biodiversity areas. In *Developed by the KBA Partnership: BirdLife International*. Gland. http://keybiodiversityareas.org/kba-data/request (last accessed date September 2023) (International Union for the Conservation of Nature, 2022).

[33] IUCN MMPATF. Global Dataset of Important Marine Mammal Areas (IUCN IMMA). [02/2023]. Made available under agreement on terms and conditions of use by the IUCN Joint SSC/WCPA Marine Mammal Protected Areas Task Force and accessible via the IMMA e-Atlas. http://www.marinemammalhabitat.org/imma-eatlas (Last Accessed July 2024) (2022).

[34] POEM- Planes de Ordenación del Espacio Marítimo. Real Decreto 150/2023, de 28 de febrero, por el que se aprueban los planes de ordenación del espacio marítimo de las cinco demarcaciones marinas españolas https://www.boe.es/diario_boe/txt.php?lang=enid=BOE-A-2023-5704 (2023).

[35] Ramirez-Llodra, E. et al. Man and the last great wilderness: human impact on the deep sea. *PLoS ONE* **6**, e22588 (2011). 21829635 10.1371/journal.pone.0022588 PMC3148232

[36] IUCN. IUCN’s Global Conservation Standards to Marine Protected Areas (MPA) Delivering Effective Conservation Action through MPAs, to Secure Ocean Health & Sustainable Development (2018).

[37] WWF-France. PHAROS4MPAs- A Review of solutions to avoid and mitigate environmental impacts of offshore windfarms. BioConsult SH on behalf of WWF France, p.264. (2019).

[38] FAO/World Bank. Biodiversity and Ecosystems Services Index: Measuring the value of nature. Swiss Re Institute, NCFA 2020, Oxford Economics 2020. https://www.swissre.com/institute/research/topics-and-risk-dialogues/climate-and-natural-catastrophe-risk/expertise-publication-biodiversity-and-ecosystems-services.html#/ (2020).

[39] Bray, L. et al. Expected effects of offshore wind farms on Mediterranean marine Life.* J. Mar. Sci. Eng*.** 4** (1), 18. 10.3390/jmse4010018 (2016).

[40] EEA 2023. Europe’s marine biodiversity remains under pressure. https://www.eea.europa.eu/publications/europes-marine-biodiversity-remains-under-pressure (last Accessed 22 May 2024).

[41] Carreño, A. & Lloret, J. environmental impacts of increasing leisure boating activity in Mediterranean coastal waters. *Ocean Coast. Manag.*10.1016/j.ocecoaman.2021.105693 (2021).

[42] D’Amore-Domenech, R., Meca, V. L., Pollet, B. G. & Leo, T. On the bulk transport of green hydrogen at sea: Comparison between submarine pipeline and compressed and liquefied transport by ship. *Energy* **15**, 126621 (2023).

[43] Hanasaki, N., Yoshikawa, S., Kakinuma, K. & Kanae, S. A seawater desalination scheme for global hydrological models. *Hydrol. Earth Syst. Sci.* **20**, 4143–4157. 10.5194/hess-20-4143-2016 (2016).

[44] Weiss, C., Guanche, R., Ondiviela, B., Castellanos, O. & Juanes, J. Marine renewable energy potential: A global perspective for offshore wind and wave exploitation. *Energy Convers. Manag.* **177**, 43–54 (2018).

[45] Crona, B. et al. Sharing the seas: a review and analysis of ocean sector interactions. *Environ. Res. Lett.* **16**, 6. 10.1088/1748-9326/ac02ed (2021).

[46] Brenner, J., Jiménez, J. A., Sardá, R. & Garola, A. An assessment of the non-market value of the ecosystem services provided by the Catalan coastal zone, Spain. *Ocean Coast. Manag.* **53**, 27–38 (2010).

[47] OECD. The Ocean Economy in 2030, 10.1787/9789264251724-en (2016).

[48] OECD. Biodiversity: Finance and the Economic and Business Case for Action. Report prepared for the G7 Environment Ministers‘ Meeting 5–6 May 2019. (2019).

[49] Béné, C. et al. Contribution of fisheries and aquaculture to food security and poverty reduction: assessing the current evidence. *World Dev.* **79**, 177–196. 10.1016/j.worlddev.2015.11.007 (2016).

[50] Niner, H. J. et al. Issues of context, capacity and scale: essential conditions and missing links for a sustainable blue economy. *Environ. Sci. Policy* **130**, 25–35 (2022).

[51] Voyer, M., Quirk, G., McIlgorm, A. & Azmi, K. Shades of blue: what do competing interpretations of the Blue Economy mean for oceans governance?. *J. Environ. Plan. Policy Manag.* **20**(5), 595–616 (2018).

[52] Pörtner, H. O., Scholes, R. J., Arneth, A., Barnes, D. K. A. & Burrows, M. T. Overcoming the coupled climate and biodiversity crises and their societal impacts. *Science* **380**, eabl4881 (2023). 37079687 10.1126/science.abl4881

[53] Supreme. Definition of the most appropriate geographical scale for MSP plans at national scale. In *Supporting Maritime Spatial Planning in the Eastern Mediterranean (SUPREME), Agreement EASME/EMFF/2015/1.2.1.3/01/S12.742087—SUPREME* (2018).

[54] Christoforou Livani, C., Cervera Núñez, C. & Smanis T. *Maritime Spatial Planning Through the Years: Insights of a Decade of EMFF and EMFAF Funded Projects*10.2926/69197 (Publications Office of the European Union, 2024).

[55] WWF. Maritime Spatial Planning in the Mediterranean Sea. Report. https://maritime-spatial-planning.ec.europa.eu/msp-resources/ec-msp-studies. (2023).

[56] Lloret, J., Carreño, A., Caric, H., San, J. & Fleming, L. E. Environmental and human health impacts of cruise tourism: A review. *Mar. Pollut. Bull.* **173**, 112979. 10.1016/j.marpolbul.2021.112979 (2021). 34598093 10.1016/j.marpolbul.2021.112979

[57] Ertör, I. & Hadjimichael, M. Editorial: Blue degrowth and the politics of the sea: rethinking the blue economy. *Sustain. Sci.* **15**, 1–10 (2020).

[58] Hadjimichael, M. A call for a blue degrowth: Unravelling the European Union’s fisheries and maritime policies. *Mar. Policy* **94**, 158–164 (2018).

[59] Pascual, U. et al. Diverse values of nature for sustainability. *Nature* **620**, 813–823. 10.1038/s41586-023-06406-9 (2023). 37558877 10.1038/s41586-023-06406-9 PMC10447232

[60] Sachs, J. *The Age of Sustainable Development*. ISBN: 9780231173155. (Columbia University Press, 2015).

[61] UNEP. Convention on Biological Diversity. Kunming-Montreal Global Biodiversity Framework. Decision CBD/COP/DEC/15/4 (2022).

[62] United Nations. The Global Goals. https://www.globalgoals.org/ (last Accessed Jan. 2025) (2015).

[63] European Commission 2020. Managing and protecting Natura 2000 sites. https://environment.ec.europa.eu/topics/nature-and-biodiversity/natura-2000/managing-and-protecting-natura-2000-sites_en (last Accessed May 2024).

[64] EEA 2025. Natura 2000 Coverage in Europe’s Seas. https://www.eea.europa.eu/themes/biodiversity/natura-2000/natura-2000-coverage-in-european-seas-4 (last Accessed 22 May 2024).

[65] Lester, S. E. et al. Biological effects within no-take marine reserves: a global synthesis. *Mar. Ecol. Prog. Ser.* **384**, 33–46 (2009).

[66] Brill, D. N., Cleary, J., Roberts, J. J., O’Brien, B. R. & Halpin, P. N. Expected occurrence of wildlife in US Atlantic offshore wind areas.* Front. Mar. Sci*.** 12**, 1602182. 10.3389/fmars.2025.1602182 (2025)

[67] Lloret, J. Offshore wind farms and marine protected areas in European waters: Better apart than together. *Mar. Pollut. Bull.* **220**, 118368. 10.1016/j.marpolbul.2025.118368 (2025). 40633151 10.1016/j.marpolbul.2025.118368

[68] EU 2014 Directive 2014/89/EU - Maritime Spatial Planning. https://www.eea.europa.eu/policy-documents/directive-2014-89-eu-maritime.

[69] Frazão Santos, C. et al. Major challenges in developing marine spatial planning. *Mar. Policy* **132**, 103248 (2021).

[70] Copping, A., Hanna, L., Grear, M. & Pomeroy, C. A framework for ecosystem-based management of marine renewable energy development. *Front. Mar. Sci.* **7**, 1–13. 10.3389/fmars.2020.00360 (2020).32802822

[71] Walsh, C., Kühl-Stenzel, A. & Schwemmer, H. Ecosystem-based maritime spatial planning in practice? On the limitations of a legalistic planning tradition. *Town Plan. Rev.* **95**(6), 643–664 (2024).

[72] Cohen, P. J. et al. Securing a just space for small-scale fisheries in the blue economy. *Front. Mar. Sci.* **6**, 1–8 (2019).36817748

[73] Evans, L. S., Buchan, P. M., Fortnam, M., Honig, M. & Heaps, L. Putting coastal communities at the center of a sustainable blue economy: A review of risks, opportunities, and strategies. *Front. Polit. Sci.* **4**, 1032204. 10.3389/fpos.2022.1032204 (2023).

[74] Bennett, N. J. et al. Towards a sustainable and equitable blue economy. *Nat. Sustain.* **2**, 991–993 (2019).

[75] Depledge, M. H. Rethinking human interactions with the oceans. *R. Soc. Open Sci.* **11**, 240808. 10.1098/rsos.240808 (2024). 39359467 10.1098/rsos.240808 PMC11444757

[76] Lloret, J. et al. The roses ocean and human health chair: A new way to engage the public in oceans and human health challenges. *Int. J. Environ. Res. Public Health* **17**(14), 5078. 10.3390/ijerph17145078 (2020). 32674437 10.3390/ijerph17145078 PMC7400534

[77] Blundo-Canto, G. et al. The different dimensions of livelihood impacts of payments for environmental services (PES) schemes: A systematic review. *Ecol. Econ.* **149**, 160–183 (2018).

[78] Torres, C. & Hanley, N. Economic valuation of coastal and marine ecosystem services in the 21st century: an overview from a management perspective. DEA WP no. 75 Working Paper Series. (2016).

[79] Lloret, J. et al. The potential benefits of water sports for health and well-being in marine protected areas: a case study in the Mediterranean. *Ann. Leis. Res.*10.1080/11745398.2021.2015412 (2021).

[80] Burkhard, B. & Gee, K. Establishing the resilience of a coastal-marine social-ecological system to the installation of offshore wind farms. *Ecol. Soc.* **17**(4), 32 (2012).

[81] Gee, K. Offshore wind power development as affected by seascape values on the German North Sea coast. *Land Use Policy* **27**, 185–194 (2010).

[82] Lloret, J. et al. Recreational and small-scale fisheries may pose a threat to vulnerable species in coastal and offshore waters of the western Mediterranean. *ICES J. Mar. Sci.* **77**, 2255–2264 (2020).

[83] Duplisea, D. E., Jennings, S., Warr, K. J. & Dinmore, T. A. A size-based model of the impacts of bottom trawling on benthic community structure. *Can. J. Fish. Aquat. Sci.* **59**, 1785–1795 (2002).

[84] Robbins, J. et al. Shipping in the north-east Atlantic: Identifying spatial and temporal patterns of change. *Mar. Pollut. Bull.* **179**, 113681. 10.1016/j.marpolbul.2022.113681 (2023). 35569289 10.1016/j.marpolbul.2022.113681

