# Supplementary material for: The new wave of ocean industrialization and the challenges for biodiversity conservation in the Mediterranean Sea: the case of the Costa Brava
Source: Sci Rep. 2025 Aug 28;15:30391. doi: 10.1038/s41598-025-15279-z (PMC12394711; doi:10.1038/s41598-025-15279-z)
Supplement: Supplementary file 1 — Supplementary Table S1. [file 41598_2025_15279_MOESM1_ESM.docx]

Supplementary Table S1. Categories of areas of marine conservation value (other than Natura 2000 protected sites= found in the study area (Costa Brava region, Mediterranean Sea, up to 12 nautical miles offshore)

| Category | Definition |
| --- | --- |
| Specially Protected Areas of Mediterranean Importance (SPAMIs) | SPAMIs are defined by the Protocol concerning Specially Protected Areas and Biological Diversity in the Mediterranean as areas of importance for conserving the components of biological diversity in the Mediterranean. Legal obligations are attached to the SPAMI status and are formulated in the binding terms of the Protocol |
| Fisheries Restricted Areas (FRAs) | FRAs are defined by national governments or the General Fisheries Commission for the Mediterranean and Black Sea as geographically-defined areas in which all or certain fishing activities are temporally or permanently banned or restricted, in order to improve the conservation and sustainable exploitation of living aquatic resources and the protection of marine ecosystems. FRAs are incorporated into governmental legislation |
| Important Bird Areas (IBAs) | IBAs are defined by BirdLife International as areas being globally important for the conservation of bird populations. |
| Important Marine Mammal Areas (IMMAs) | IMMAs are defined by the Marine Mammal Protected Areas Task Force as discrete portions of habitat, important to marine mammal species, that have the potential to be delineated and managed for conservation |
| Key Biodiversity Areas (KBAs) | KBAs are defined by a partnership of global conservation organizations as the most important places in the world for species and their habitats |
