# Supplementary material for: The new wave of ocean industrialization and the challenges for biodiversity conservation in the Mediterranean Sea: the case of the Costa Brava
Source: Sci Rep. 2025 Aug 28;15:30391. doi: 10.1038/s41598-025-15279-z (PMC12394711; doi:10.1038/s41598-025-15279-z)
Supplement: Supplementary file 2 — Supplementary Table S2. [file 41598_2025_15279_MOESM2_ESM.docx]

Supplementary Table S2. Detailed information on data sources used for each industrial activity analyzed

| **Activity** | **Variable**  **(units)** | **Temporal coverage** | **Spatial coverage** | **Data source** | **Comments** |
| --- | --- | --- | --- | --- | --- |
| Fisheries and aquaculture | Seafood landed  (tons) | 2000-2023 | Fishing ports of Llançà, El Port de la Selva, Cadaqués, Roses, L’Escala, l’Estaratit, Palamós, Sant Feliu de Guíxols, Blanes | Autonomous Government of Catalonia  https://agricultura.gencat.cat/ca/ambits/pesca/dar_estadistiques_pesca_subhastada/index.html | The landings statistics are the official figures reported by the regional government. |
|  | Number of fishing vessels | 2000-2023 | Fishing ports of Llançà, El Port de la Selva, Cadaqués, Roses, L’Escala, l’Estaratit, Palamós, Sant Feliu de Guíxols, Blanes | Autonomous Government of Catalonia  https://agricultura.gencat.cat/ca/ambits/pesca/dar_estadistiques_pesca_subhastada/index.html | The number of fishing vessels are the official figures reported by the regional government. |
|  | Revenues generated by landings  (thousand Euros) | 2000-2023 |  | Autonomous Government of Catalonia  https://agricultura.gencat.cat/ca/ambits/pesca/dar_estadistiques_pesca_subhastada/index.html | The revenues are official figures reported by the regional government. The data have been adjusted for inflation based on the Spanish Consumer Price Index, as provided by the Spanish Statistics Institute (https://www.ine.es/en/) |
|  | Aquaculture sites | 2023 | Costa Brava | Autonomous Government of Catalonia  https://agricultura.gencat.cat/ca/serveis/cartografia-sig/bases-cartografiques/pesca-maritima-proteccio-litoral/installacions-aquicultura/index.html | Aquaculture sites are based on official data provided by the regional government |
| Transport of goods | Total gross weight (tonnes) | 2000-2023 | Port of Palamós | Statistical yearbooks of Ports de la Generalitat  <https://ports.gencat.cat/dades-economiques-i-anuari-estadistic/>  https://www.idescat.cat/ | Goods transported into and out of the Costa Brava ports provided by the regional government |
| Passenger traffic | Number of passengers | 2000-2023 | Large cruises: ports of Palamós and Roses  Small cruises: Ports of Port de la Selva, Roses l'Escala, l'Estartit, Palamós and Blanes | Statistical yearbooks of Ports de la Generalitat  <https://ports.gencat.cat/dades-economiques-i-anuari-estadistic/>  <https://www.idescat.cat/> | Total number of passengers embarking or disembarking on local and international cruises at ports provided by the regional government |
| Recreational boating activity | Number of berths in Costa Brava marinas | 2000-2023 | Marinas of Portbou, Colera, Llançà, El Port de la Selva, Roses/Sta. Margarida, Empúriabrava, l’Escala l’Estartit, Aiguablava/Llafranch, Palamós/Marina Palamós, Port d’Aro, St. Feliu de Guíxols, Cala Canyelles, Blanes | Statistical yearbooks of Ports de la Generalitat  <https://ports.gencat.cat/dades-economiques-i-anuari-estadistic/>  <https://www.idescat.cat/> | Marina sites provided by the regional government. |
| Economic port activity | Total port revenues (thousand Euros) | 2000-2023 | Ports of Llançà, El Port de la Selva, Roses, l'Escala, l'Estartit, Palamós, Sant Feliu de Guíxols, Blanes | Statistical yearbooks of Ports de la Generalitat  <https://ports.gencat.cat/dades-economiques-i-anuari-estadistic/>  <https://www.idescat.cat/> | Total port revenues generated by fisheries and aquaculture, transport of good, passenger traffic and recreational activity. These revenues include fees, taxes, and licenses. Data provided by the regional government. The data have been adjusted for inflation based on the Spanish Consumer Price Index, as provided by the Spanish Statistics Institute (https://www.ine.es/en/) |
| Vessel traffic | Route densities: Average presence of vessels  (hours in a Km^2^ per month) | 2023 averages | Costa Brava | Emodnet (European Marine Observation and Data Network Human Activities web portal) https://emodnet.ec.europa.eu/en/human-activities | Route densities of different vessel types (fishing vessels, cargo vessels, tankers, and cruises)  Data collected from the Automatic Identification System (AIS)  Only those ship types labelled as fishing, passenger, cargo, and tanker were analyzed. It was not possible to create a route density map of recreational vessels because most of them, due to their size, are not obliged to carry AIS devices |
| Offshore wind energy | Offshore wind development areas | 2023 | Costa Brava | Spanish Ministry for Ecological Transition and Demographic Challenge  https://ww.mapama.gob.es/ide/metadatos/srv/spa/catalog.search#/home | Data are based on the Royal Decree 150/2023, of 28 February, which approved the Spanish Maritime Spatial Plan |
| Offshore wind energy | Planned cable route | 2023 | Costa Brava | IREC  https://www.irec.cat/es/research/strategic-initiatives/plemcat/ | This is the route of the cable exporting energy from the offshore wind farms to the land. It was drawn from plans for the pilot offshore wind farm “PLEMCAT” |
| Hydrogen pipeline | Planned submarine hydrogen pipeline | 2023 | Costa Brava | https://www.elespanol.com/invertia/empresas/energia/20240625/enagas-participacion-inicial-barmar-corredor-hidrogeno-barcelona-marsella/865663792_0.html | Planned submarine hydrogen pipeline linking Spain and France, more specifically Barcelona and Marseille (the so-called H2Med project).  The pipeline layout was inferred from grey literature, as there is no official information available. |
| Seawater desalination | Desalination plants | 2023 | Costa Brava | Catalan Water Agency https://aca.gencat.cat/ca/laigua/infraestructures/dessalinitzadores/index.html | Data provided by the regional government. |
