# Supplementary material for: The new wave of ocean industrialization and the challenges for biodiversity conservation in the Mediterranean Sea: the case of the Costa Brava
Source: Sci Rep. 2025 Aug 28;15:30391. doi: 10.1038/s41598-025-15279-z (PMC12394711; doi:10.1038/s41598-025-15279-z)
Supplement: Supplementary file 3 — Supplementary Table S3. [file 41598_2025_15279_MOESM3_ESM.docx]

Supplementary Table S3. Summary of the potential environmental effects of the five industrial sectors operating in the Costa Brava area (North-western Mediterranean Sea) on Natura 2000 protected sites and other areas of conservation value, in relation to the 11 Good Environmental Status (GES) descriptors of the Marine Strategy Framework Directive^^[[1]](#footnote-1)^^.

| **GES Descriptor** | **Industrial sector** | **Potential effects** | **Key references** |
| --- | --- | --- | --- |
| 1 Biodiversity | Fisheries and aquaculture | Professional and recreational fishing gear (trawls, long-lines, etc.) with effects on target species and benthic habitats, some of which are protected or endangered species and habitats.  Bycatch of sensitive species (birds, sharks, turtles, mammals, etc.) by recreational and professional fisheries  Lost or abandoned ﬁshing gear causes harm because it continues to catch fish.  Risks of entanglement of animals in fish farms.  Overfeeding in fish farms which may drift into surrounding foodwebs and favour certain organisms over others, thereby causing changes in benthic community structure | Bolognini et al. (2019), Piante et al (2019); Higueruelo et al (2023); Purroy (2010), Domínguez Carrió et al. (2022), Indemares (2013), Intemares (2023), García de Vinuesa (2020), Lloret et al. (2008a), Mallol (2005), Indemares (2012), Gómez et al (2019). |
|  | Maritime transport of goods | Physical disturbance, noise, collisions and chemical pollution (antifouling paint, hydrocarbons, other toxic compounds, and macrowaste) threatens the survival of many species.  Sediment resuspension in shallow areas, potentially threatening seagrass meadows and benthic fauna | Abdulla and Linden (2008), Randone et al. (2019) |
|  | Maritime tourism | Effects of anchoring from leisure boating.  Impacts associated with oil pollution, black and grey waters, macrowaste, the use of antifouling paints. Collisions and noise generated by the vessel’s engines. Sediment resuspension in shallow areas, potentially threatening seagrass meadows and benthic fauna | Caric et al. (2019), Carreño and Lloret (2021), Lloret et al. (2021), Carreño et al. (2019) |
|  | Marine energy | Offshore wind farms can lead to the loss of fragile benthic habitats, disturbance to sensitive and threatened species, alteration of species composition, collision and entanglement of marine mammals, risk of accidents and artificial reef effect  The impact of the deployment of pipelines on biodiversity has been little studied, with mixed results (neutral or negative impacts have been observed) | WWF-France (2019), Lloret et al. (2022, 2023), Wawrzynkowski et al. (2025), Cotugno et al. (2020), Spagnolo et al. (2019) |
|  | Desalination | No significant variations attributable to the brine discharges from a desalination plant were found | Raventós et al. (2006) |
| 2 Non-indigenous species | Fisheries and aquaculture | Live exotic baits such as worms used in recreational angling in coastal MPAs represent an increasing environmental threat. Potential escapes and introduction of non-indigenous species from aquaculture sites | Bolognini et al. (2019), Font and Lloret (2008), Gómez et al (2019) |
|  | Maritime transport of goods | Spread of invasive organisms (including pathogens) through ballast water discharge. | Abdulla and Linden (2008), Randone et al. (2019) |
|  | Maritime tourism | Spread of invasive organisms (including pathogens) through ballast water discharge. | Caric et al. (2019), Carreño and Lloret (2021), Lloret et al. (2021), Carreño et al. (2019) |
|  | Marine energy | The artificial substrates created by the offshore wind farms may favor the colonization of non-indigenous species  The impacts of hydrogen pipelines are unknown | WWF-France (2019), Lloret et al. (2022, 2023) |
|  | Desalination | Unknown |  |
| 3 Commercial fish and shellfish | Fisheries and aquaculture | Effects of professional and recreational fisheries on exploited species, particularly threatened or protected species  In aquaculture, interactions with wild commercial species through accidental escape of farmed fish; involuntary release of gametes/larvae; exchange of parasites and pathogens or release of cultured fish for restocking | ICATMAR (2024 a,b), Lloret et al. (2008a), Lloret and Riera (2008), Bolognini et al. (2019), Piante et al. (2019), Gómez et al (2019) |
|  | Maritime transport of goods | Unknown |  |
|  | Maritime tourism | Unknown |  |
|  | Marine energy | There may be effects of offshore wind farms on exploited species due to sound, vibrations and electromagnetic fields from cables  The impacts of hydrogen pipelines are unknown | Lloret et al. (2022, 2023) |
|  | Desalination | Unknown |  |
| 4 Food webs | Fisheries and aquaculture | Some professional and recreational fishing methods target high trophic level fish, which may lead to changes in the food webs (cascading effects of loss of predators on prey species)  In aquaculture, effects on predator control due to fish-eating birds, marine mammals and other fish | Corrales et al. (2020), Bolognini et al. (2019), Piante et al. (2019), Gómez et al. (2019) |
|  | Maritime transport of goods | Unknown |  |
|  | Maritime tourism | Unknown |  |
|  | Marine energy | Colonization by new communities on offshore wind farms that can modify food webs and biogeochemical cycling  The impacts of hydrogen pipelines are unknown | WWF-France (2019), Lloret et al. (2022, 2023) |
|  | Desalination | Unknown |  |
| 5 Eutrophication | Fisheries and aquaculture | In aquaculture, nitrogen and phosphorus load from waste material; trace elements and dissolved organic matter load from decomposition of uneaten feed and fecal material | Bolognini et al. (2019) |
|  | Maritime transport of goods | Sediment resuspension in shallow areas, potentially threatening seagrass habitats and benthic fauna |  |
|  | Maritime tourism | Motor vessels (leisure boats and cruises) with propeller engines may generate sediment suspension in shallow waters, contributing to the turbidity of the water and promoting eutrophication | Carreño et al. (2019), Caric et al. (2019), Carreño and Lloret (2021); Lloret et al. (2021) |
|  | Marine energy | Unknown |  |
|  | Desalination | Unknown |  |
| 6 Sea-floor integrity | Fisheries and aquaculture | Professional fishing gears affect benthic assemblages, particularly structural sessile organisms  In aquaculture, turbidity and changes in dissolved O2 and nutrients can affect certain coastal habitats, such as seagrass meadows and maerl beds | Higueruelo et al. (2023), Purroy (2010), Domínguez Carrió et al. (2022), Indemares (2013), García de Vinuesa (2020), Mallol (2005), Piante et al. (2019), Bolognini et al. (2019) |
|  | Maritime transport of goods | Damage to benthic habitats and increase of turbidity leading to alteration of sea-floor integrity | Abdulla and Lilnden, (2008), Randone et al. (2019) |
|  | Maritime tourism | Anchors from recreational boats and cruises can affect the integrity of the sea-floor (e.g. seagrass meadows) | Caric et al. (2019), Carreño et al. (2019), Carreño and Lloret (2021), Lloret et al. (2021) |
|  | Marine energy | Potential habitat alterations due to the infrastructure (anchors, chains and export cables) related to offshore wind farms  The impact of the deployment of pipelines on marine habitats has been little studied, with mixed results (neutral or negative impacts have been observed) | WWF-France (2019), Spagnolo et al. (2019), Cotugno et al. (2020), Lloret et al. (2022, 2023) |
|  | Desalination | Unknown |  |
| 7 Hydrographical conditions | Fisheries and aquaculture | Unknown |  |
|  | Maritime transport of goods | Unknown |  |
|  | Maritime tourism | Unknown |  |
|  | Marine energy | Offshore wind farms can lead to alterations in stratification patterns and wind fields, leading to changes in local primary productivity, carbon ﬂow to the benthos, and changes in larval transport pathways  The impacts of hydrogen pipelines are unknown | WWF-France (2019), Lloret et al. (2022, 2023) |
|  | Desalination | Extraction of freshwater from the nearby river aquifers | Otero et al. (2011) |
| 8 Contaminants in the marine environment | Fisheries and aquaculture | Engine emissions and chemical pollution from antifouling paints used in professional and recreational fishing vessels  Abandoned, lost or discarded professional and recreational fishing gear and fishing tackle.  Effluent discharges from aquaculture facilities may contain residues of veterinary products (disinfectants and drugs for disease treatments) and antifouling agents | Consoli et al. (2018, 2019), Bolognini et al. (2019), Piante et al. (2019), Gómez et al (2019). |
|  | Maritime transport of goods | Regular dispersion of oil and other contaminants from antifouling and ballast waters, and accidental pollution (e.g. oil spills) | Abdulla and Linden (2008), Randone et al. (2019) |
|  | Maritime tourism | Contamination from toxic antifouling products, engine emissions and discharge of bilge waters by leisure boats and cruises, among others | Carreño et al. (2019), Caric et al. (2019), Carreño and Lloret (2021), Lloret et al. (2021) |
|  | Marine energy | Offshore wind farms can pollute waters because of leaks, toxic antifouling agents, and accidents  The impacts of hydrogen pipelines are unknown | WWF-France (2019), Lloret et al. (2022, 2023) |
|  | Desalination | Unknown |  |
| 9 Contaminants in seafood | Fisheries and aquaculture | Unknown |  |
|  | Maritime transport of goods | Unknown |  |
|  | Maritime tourism | Unknown |  |
|  | Marine energy | Unknown |  |
|  | Desalination | Unknown |  |
| 10 Marine litter | Fisheries and aquaculture | Marine litter related to gear and tackle lost at sea (lines, pots, nets, hooks and weights) by professional and recreational fishers and aquaculture | Higueruelo et al. (2023); Consoli et al. (2018, 2019), Bolognini et al. (2019), Piante et al. (2019), Gómez et al (2019). |
|  | Maritime transport of goods | Loss or discharge of marine litter | Abdulla and Linden (2008); Randone et al. (2019) |
|  | Maritime tourism | Plastics and other litter dumped by recreational boats and cruises | Caric et al. (2019), Carreño and Lloret (2021), Lloret et al. (2021) |
|  | Marine Energy | Unknown |  |
|  | Desalination | Unknown |  |
| 11 Energy, including Underwater Noise | Fisheries and aquaculture | Unknown |  |
|  | Maritime transport of goods | Underwater noise affecting communication, predator-prey interactions and biology (growth and reproduction) of marine species | Abdulla and Linden (2008), Randone et al. (2019) |
|  | Maritime tourism | Underwater noise (from recreational motor craft and cruises) affecting communication, predator-prey interactions and biology (growth and reproduction) of marine species | Caric et al. (2019), Carreño et al. (2019); Carreño and Lloret (2021), Lloret et al (2021) |
|  | Marine energy | Significant marine noise and vibration from wind turbines and mounting structures. Emission of electromagnetic fields (cables)  The impacts of hydrogen pipelines are unknown | WWF-France (2019), Lloret et al. (2022, 2023) |
|  | Desalination | Unknown |  |

**Bibliography**

Abdulla, A., Linden, O. (editors). 2008. Maritime traffic effects on biodiversity in the Mediterranean Sea: Review of impacts, priority areas and mitigation measures. Málaga, Spain: IUCN Centre for Mediterranean Cooperation. 184 pp.

Ballesteros, E. 2004. L’entorn marí de Tossa de Mar, Lloret de Mar i Blanes. Ed. Competium. 4, 124 pp

Bolognini, L., Grati, F., Marino, G., Punzo, E., Scanu, M., Torres, C., Hardy, P.Y., Piante, C. 2019. Safeguarding Marine Protected Areas in the growing Mediterranean Blue Economy. Recommendations for Aquaculture. PHAROS4MPAs project. 52 pages.

Caric, H., Jakl, Z., Laurent, C., Mackelworth, P., Noon, V., Petit, S., Piante, C., Randone, M. 2019. Safeguarding Marine Protected Areas in the growing Mediterranean Blue Economy. Recommendations for the cruise sector. PHAROS4MPAs project report. 48 pages.

Carreño, A., Hardy, P.-Y., Sánchez, E., Martínez, E., Piante C, Lloret, J. 2019. Safeguarding Marine Protected Areas in the growing Mediterranean Blue Economy. Recommendations for Leisure Boating. PHAROS4MPAs project. 52 pages.

Carreño, A., Lloret, J. 2021. Environmental impacts of increasing leisure boating activity in Mediterranean coastal waters. Ocean & Coastal Management. Elsevier BV. http://doi.org/10.1016/j.ocecoaman.2021.105693

Consoli P, Falautano M, Sinopoli M, Perzia P, Canese S, Esposito V, Battaglia P, Romeo T, Andaloro F, Galgani F, Castriota L. 2018. Composition and abundance of benthic marine litter in a coastal area of the central Mediterranean Sea. Mar. Pollut. Bull. 136:243-247

Consoli P, Romeo T, Angiolillo M, Canese S, Esposito V, Salvati E, Scotti G, Andaloro F, Tunesi L. 2019. Marine litter from fishery activities in the Western Mediterranean Sea: The impact of entanglement on marine animal forests. Environ Pollut. 249:472-481.

Corrales, X., Vilas, D., Piroddi, C., Steenbeek, J., Claudet, J., Lloret, J., et al. 2020. Multi-zone marine protected areas: Assessment of ecosystem and fisheries benefits using multiple ecosystem models. Ocean & Coastal Management 193: 105232. http://doi.org/10.1016/j.ocecoaman.2020.105232

Cotugno, M., Lorenti, M., Scipione, M.B., Patti, F.P., Buia, M.C. 2020. Laying a gas pipeline through a Posidonia oceanica meadow: an example of its effects on plant recovery and epifaunal diversity. Vie et milieu - Life and environment 70 (3-4): 63-68

Dominguez-Carrió, C., Riera, J. L., Robert, K., Zabala, M., Requena, S., Gori, A., et al. 2022. Diversity, structure and spatial distribution of megabenthic communities in Cap de Creus continental shelf and submarine canyon (NW Mediterranean). Progress in Oceanography. http://doi.org/10.1016/j.pocean.2022.102877

Font, T., Gil, J., Lloret, J. 2018. The commercialization and use of exotic baits in recreational fisheries in the north-western Mediterranean: Environmental and management implications. Aquatic Conservation: Marine and Freshwater Ecosystems 28(3): 651-661

García de Vinuesa, A. 2020. Evaluación de la vulnerabilidad y del estado de conservación de ecosistemas marinos bentónicos especialmente productivos del Mediterráneo frente al impacto de la pesca de arrastre, para impulsar su correcta gestión. PhD. Thesis, University of Barcelona

Gómez, S., Carreño, A., Sánchez, E., Martínez, E., Lloret, J. 2019. Safeguarding Marine Protected Areas in the growing Mediterranean Blue Economy. Recommendations for Recreational Fisheries. PHAROS4MPAs project. 56 pages.

Higueruelo, A, Santín A, Salazar J, Ambroso S, Soler-Membrives A, Grinyó J. 2023 Coexistence of megabenthic assemblages and artisanal fishers: The case of Cap de Creus Marine Protected Area (North-Western Mediterranean Sea). Mar Environ Res. 2023 Nov;192:106211. doi: 10.1016/j.marenvres.2023.106211. Epub 2023 Oct 1. PMID: 37801780.

ICATMAR 2024a State of fisheries in Catalonia 2023, Part 1: Report on the monitoring of the commercial fishing fleet (ICATMAR, 24-05) https://www.icatmar.cat/publicacions/ https://www.icatmar.cat/publicacions/

ICATMAR 2024b. State of Marine Recreational Fisheries in Catalonia 2023 (ICATMAR, 24-04) https://www.icatmar.cat/publicacions/

Indemarees 2012. Directrices de gestión y seguimiento ZEPA ES0000514 Espacio marino de l’Empordà. Melissa Consultoría e Ingeniería ambiental. 23pp. https://www.indemares.es/sites/default/files/_0613apendice_mediterraneo_es0000514_emdel_emporda.pdf

Indemares 2013. Análisis de la huella pesquera anexo a la memoria justificativa de la zonificación propuesta para la zona Indemares. https://www.indemares.es/sites/default/files/analisis_huella_pesquera_golfo_leon_csic.pdf

Intemares 2023. Dosier informativo taller de participación para la elaboración del plan de gestión del LIC sistema de cañones submarinos occidentales del golfo de León (ESZZ16001). https://intemares.es/wp-content/uploads/2023/03/inf_golfo_de_leon.pdf

Lloret, J., Riera, V. 2008. Evolution of a Mediterranean Coastal Zone: Human Impacts on the Marine Environment of Cape Creus. Environmental Management. Springer Science and Business Media LLC. http://doi.org/10.1007/s00267-008-9196-1

Lloret, J., Zaragoza, N., Caballero, D., Font, T., Casadevall, M., & Riera, V. 2008. Spearfishing pressure on fish communities in rocky coastal habitats in a Mediterranean marine protected area. Fisheries Research. Elsevier BV. http://doi.org/10.1016/j.fishres.2008.07.002

Lloret, J., Carreño, A., Caric, H., San, J., Fleming, L.E. 2021 Environmental and Human Health Impacts of Cruise Tourism: a review. Marine Pollution Bulletin 173, 112979, 10.1016/j.marpolbul.2021.112979

Lloret J, Turiel A, Solé J, Berdalet E, Sabatés A, Olivares A, Gili JM, Vila-Subirós J, Sardá R. 2022 Unravelling the ecological impacts of large-scale offshore wind farms in the Mediterranean Sea. Sci Total Environ. 10;824:153803. doi: 10.1016/j.scitotenv.2022.153803

Lloret, J., Wawrzynkowski, P., Dominguez-Carrió, C., Sardá, R., Molins, C., Gili, J. M., et al 2023. Floating offshore wind farms in Mediterranean marine protected areas: a cautionary tale. ICES Journal of Marine Science. http://doi.org/10.1093/icesjms/fsad131

Mallol, S. 2005. Anàlisi dels descartaments efectuats per la flota d’arrossegament en el Golf de Lleó . PhD Thesis. Universitat de Girona. 281 pp. (in Catalan)

Otero, N.; Soler, A.; Corp, R.M.; Mas-Pla, J.; Garcia-Solsona, E.; Masqué, P. 2011. Origin and evolution of groundwater collected by a desalination plant (Tordera, Spain): a multi-isotopic approach. Journal of Hydrology 397: 37-46.

Piante C., Kapedani R., Hardy, P.-Y., Gallon S. 2019. Safeguarding Marine Protected Areas in the growing Mediterranean Blue Economy. Recommendations for Small-Scale Fisheries. PHAROS4MPAs project. 52 pages.

Purroy, A. 2010. Spatial assessment and impact of artisanal fisheries’ activity in Cap de Creus. Master thesis submitted for the partial fulfillment of the title of Master of Science in Marine Biodiversity and Conservation Within the Erasmus Mundus Master Programme EMBC. 95 pp.

Randone, M., Bocci, M., Castellani, C.,Laurent, C. 2019. Safeguarding Marine Protected Areas in the growing Mediterranean Blue Economy. Recommendations for Maritime Transport. PHAROS4MPAs project. 64 pages

Raventós, N., Macpherson, E., & García-Rubiés, A. 2006. Effect of brine discharge from a desalination plant on macrobenthic communities in the NW Mediterranean. Marine Environmental Research. Elsevier BV. http://doi.org/10.1016/j.marenvres.2006.02.00

Spagnolo A, Cuicchi C, De Biasi AM, Ferrà C, Montagnini L, Punzo E, Salvalaggio V, Santelli A, Strafella P, Fabi G. 2019. Effects of the installation of offshore pipelines on macrozoobenthic communities (northern and central Adriatic Sea). Mar Pollut Bull. 138:534-544.

Wawrzynkowski, P.; Molins, C.; Lloret, J. 2025 Assessing the potential impacts of floating Offshore Wind Farms on policy-relevant species: A case study in the Gulf of Roses, NW Mediterranean. Marine Policy 172, 106518.

WWF-France 2019. Safeguarding marine protected areas in the growing Mediterranean blue economy. Recommendations for the offshore wind energy sector. PHAROS4MPAs project. 68 pages

1. Biodiversity: The quality and occurrence of habitats and the distribution and abundance of species are in line with prevailing physiographic, geographic and climatic conditions. Non-indigenous species: Non-indigenous species introduced by human activities are at levels that do not adversely alter the ecosystems. Commercial fish and shellfish: populations of all commercially exploited fish and shellfish are within safe biological limits, exhibiting a population age and size distribution that is indicative of a healthy stock. Food webs: all elements of the marine food webs, as far as they are known, occur at normal abundance and diversity and at levels capable of ensuring the long-term abundance of the species and the retention of their full reproductive capacity. Eutrophication: human-induced eutrophication is minimised, and especially its adverse effects, such as biodiversity losses, ecosystem degradation, harmful algae blooms and oxygen deficiency in bottom waters. Sea-floor integrity: Sea-floor integrity is at a level that ensures that the structure and functions of the ecosystems are safeguarded and benthic ecosystems in particular are not adversely affected. Hydrographical conditions: Permanent alteration of hydrographical conditions does not adversely affect marine ecosystems. Contaminants in the marine environment: Contaminants are at a level not giving rise to pollution effects. Contaminants in seafood: contaminants in fish and other seafood for human consumption do not exceed levels established by Community legislation or other relevant standards. Marine litter: properties and quantities of marine litter do not cause harm to the coastal and marine environment. Energy, including Underwater Noise: Introduction of energy, including underwater noise, is at levels that do not adversely affect the marine environment. [↑](#footnote-ref-1)
